# Paediatric anaemia prevalence trends in the Eastern Mediterranean Region: a 20-year analysis by income level

**DOI:** 10.7189/jogh.15.04160

**Published:** 2025-05-05

**Authors:** Reem Eltayeb, Naif K Binsaleh, Husam Qanash, Reem M Ali, Nagwan Elhussein, Mona HM Ahmed

**Affiliations:** 1 Department of Medical Laboratory Science, College of Applied Medical Science, University of Ha’il, Ha’il, Saudi Arabia; 2 Medical and Diagnostic Research Center, University of Ha’il, Ha’il, Saudi Arabia; 3 Department of Diagnostic Radiology, College of Applied Medical Sciences, University of Ha’il, Ha’il, Saudi Arabia; 4 Department of Nutrition, College of Applied Medical Sciences, University of Ha’il, Ha’il, Saudi Arabia

## Abstract

**Background:**

Anaemia is a significant global public health issue, especially in low-income countries, where it influences children’s mental and physical development. Anaemia is a common public health problem in the Eastern Mediterranean Region (EMR), although long-term, income-stratified analyses at the regional level are scarce. This study investigated trends in anaemia prevalence among children aged 6–59 months from 21 countries in the EMR between 2000 and 2019, correlating differences in these trends with each country’s national income.

**Methods:**

We analysed data collected by the World Health Organization (WHO) on the prevalence of anaemia in children aged 6–59 months between 2000 and 2019 in 21 EMR countries at five-year intervals. Data on national income classifications were taken from the World Bank. The data were subject to statistical analysis using SPSS, Excel and GraphPad Prism to determine the associations between anaemia prevalence and income groups.

**Results:**

The average anaemia prevalence among children in the EMR decreased from 40.56% in 2000 to 34.25% in 2019 – an absolute change of −6.3%. The number of countries with a prevalence above 40% fell from seven in 2000 to six in 2019. Oman, Yemen and Sudan had some of the highest prevalences, and the UAE witnessed a slight increase, despite being a high-income country. Anaemia prevalence was significantly associated with national income (*P* < 0.001), but outliers suggest that income alone does not fully explain the observed trends. In 2019, the prevalence of anaemia in the EMR remained greater than the global average.

**Conclusions:**

Between 2000 and 2019, the prevalence of anaemia in children aged 6–59 months decreased significantly in the EMR. Despite this progress, there are still disparities between the prevalence of anaemia in low-income and high-income countries. Therefore, income is not the only factor associated with declines in anaemia prevalence. The fact that reduction rates plateaued after 2015 reinforces the significance of implementing new, innovative health programmes to minimise anaemia.

Anaemia is a common global public health issue that particularly impacts paediatric populations, especially in low- and lower-middle-income countries [1]. It is a condition characterised by a reduced concentration of haemoglobin (Hb) or number of red blood cells [2]. The reduced levels of Hb restrict the transfer of oxygen in the blood, leaving the blood flow unable to fulfil the body’s physiological requirements [3]. This leads to diminished physical and mental capacity, accompanied by greater susceptibility to various adverse health consequences [4].

The World Health Organization (WHO) defines anaemia in children aged 6 − 59 months as an Hb concentration of less than 110 grammes per litre (g/L), adjusted for altitude [5]. The gravity of anaemia as a public health issue is evident from the frequency of its occurrence in various regions. Anaemia is considered to be (a) not a public health issue when ≤4.9% of the population is affected, while it is considered (b) mild when 5.0–19.9% of the population is impacted, (c) moderate when 20.0–39.9% of the population is afflicted, and (d) severe when ≥40.0% of the population is affected [6].

Anaemia is frequently a multifactorial condition involving interrelated interactions of nutritional and non-nutritional factors and mechanisms [7]. It is induced by one’s diet, infection burden, access to health care and socioeconomic environment. In the Eastern Mediterranean Region (EMR) – a region embracing countries with marked variation in social, economic, political and developmental contexts – anaemia is still common among children [8]. The EMR has consistently reported higher anaemia prevalences than the global average, yet it remains underrepresented in comparative trend analyses [9,10]. Nevertheless, there have been limited investigations into long-term, multi-country patterns within the region, and such investigations have rarely compared countries by income group or assessed temporal trends in detail.

Multiple initiatives have been launched to address this regional issue. The Regional Nutrition Strategy 2010–2019 and Action Plan aimed to decrease the prevalence of iron-deficiency anaemia by 30% [11]. More recently, the Eastern Mediterranean Nutrition Strategy 2020–2030 was issued by the World Health Assembly to reduce the prevalence of anaemia in the region [12]. However, few peer-reviewed studies have evaluated the effectiveness of these initiatives or documented regional progress in anaemia reduction across countries with different income levels [13].

To fill this gap, the current study had two aims:

1) to examine changes in the prevalence of anaemia among children aged 6 to 59 months in 21 countries in the EMR

2) to provide insights into variations in anaemia trends from 2000 to 2019, measured at five-year intervals. By linking anaemia prevalence to national income classification, this study explores the relationship between economic development and child health outcomes in a region marked by disparities in both categories.

In our earlier work, we investigated how the global prevalence of anaemia in children under five correlates with national income [14]. However, this prior analysis lacked geographic specificity and did not allow for an in-depth understanding of local or regional variations. In this study, we overcome that limitation by focusing exclusively on the EMR and stratifying trends by country and income level over a 20-year period.

The hypothesis guiding the study was that the prevalence of anaemia among children in the EMR is strongly associated with country income level, but socioeconomic and geopolitical factors – such as conflict, health system capacity and nutrition programmes – may generate heterogeneity within income groups. This hypothesis enabled a targeted exploration of how income-related disparities shape the paediatric anaemia burden across a single global region.

## METHODS

This research used the same methods as our previous study [14] except that, in the current study, the object of our study was anaemia prevalence in the EMR region. We analysed data from 21 countries across the EMR to identify trends in anaemia prevalence among children aged 6–59 months between 2000 and 2019. The data were gathered from the WHO’s Information System Database.

### Inclusion criteria

We included data on anaemia prevalence if they were publicly available on the WHO’s website, if full data were supplied for the period 2000–2019 and if the website classified each country’s income in 2022.

### Exclusion criteria

Countries were excluded if they did not have complete WHO-reported anaemia prevalence data covering the years 2000–2019, ensuring consistency in the trend analysis. Additionally, countries lacking World Bank income classifications in 2022 were excluded to maintain uniform socioeconomic categorisations. Subnational or regional-level data were not considered, as this study focused on national-level prevalence trends. Furthermore, data sets that used definitions of anaemia differing from the WHO standard (Hb <110 g/L for children aged 6–59 months, adjusted for altitude) were excluded to uphold methodological consistency. Notably, all 21 countries included in the study met these criteria, and no missing data were identified within the analysed data set.

### Data sources

The data analysed in this study were obtained from the Global Health Observatory of the WHO and the World Bank database. Although annual data were available, we grouped the data into five-year intervals to reduce year-to-year fluctuations, improve the interpretability of long-term trends and facilitate comparisons across countries and income groups. This approach also aligns with practices in global health reporting, where five-year benchmarks are commonly used for trend analysis and policy evaluation. The data set was subjected to rigorous quality control procedures by the WHO to ensure its accuracy and comparability. While estimations and modelling approaches may be used by the WHO in cases in which direct survey data are unavailable, we verified that no missing data or gaps were present in our data set.

The data collected from the WHO can be found on the following website:

https://www.who.int/data/gho/data/indicators/indicator-details/GHO/prevalence-of-anaemia-in-children-under-5-years-(-)

The World Bank’s classification of national incomes can be found at the following address: https://datahelpdesk.worldbank.org/knowledgebase/articles/906519-world-bank-country-and-lending-groups.

### Analytical phase

Our analysis consisted of four steps. The first part involved locating, analysing and collecting data on the prevalence of anaemia. The second phase entailed linking this prevalence to each country’s income as classified by the World Bank in 2022. The income levels are divided into four categories: low (1045 USD or less), lower middle (1046 to 4095 USD), higher middle (4096 to 12 695 USD) and high income (12 696 USD or above). Income group assignments were based on the World Bank’s 2022 income classification to provide a unified and current framework for the analysis and avoid inconsistencies caused by shifting country categories over time.

The third phase involved examining trends in anaemia prevalence across four five-year periods (2000–2005, 2005–2010, 2010–2015 and 2015–2019) and correlating them with income levels. We used descriptive statistics to summarise anaemia prevalence over time, and we tested the normality assumptions using the Kolmogorov-Smirnov and Shapiro-Wilk tests. The Kruskal-Wallis test was selected because the data were not normally distributed and involved comparisons across more than two independent income groups. We considered a two-tailed *P*-value of <0.05 to indicate statistical significance. Additional thresholds of <0.01 and <0.001 are noted where relevant to emphasise highly significant findings. As the focus of this study was primarily descriptive and exploratory, formal multiple testing correction methods were not applied.

In the fourth phase, we estimated changes in anaemia prevalence across five-year intervals and compared them based on their relative variations. We also compared trends in the EMR with global trends to identify divergences and commonalities.

In this study, a small decrease in anaemia prevalence was defined as a relative drop of less than 3% over two decades, while a large drop was defined as a relative decline of more than 10%. These thresholds were chosen based on the interquartile range distribution observed in the literature on global anaemia trends, including WHO and Global Burden of Disease (GBD) data, and they are consistent with previous classifications of modest *vs*. substantial public health improvements [15,16].

## RESULTS

This section highlights key findings from our analysis of paediatric anaemia trends in 21 EMR countries between 2000 and 2019. The results are organised to emphasise broad trends before breaking down country- and income-level details.

### Key highlights

The mean prevalence of children with anaemia in the EMR decreased from 40.56% in 2000 to 34.25% in 2019, representing an absolute decrease of 6.31% and a relative decline of 16%. The prevalence of anaemia was consistently highest in low-income countries, with six countries reporting prevalences above 40% in 2019. We found statistically significant associations between national income level and prevalence of anaemia (*P* < 0.001). The largest relative decrease among low-income countries occurred in Somalia, but a small increase occurred in the UAE, a high-income country. Upper-middle-income countries achieved the largest reductions overall.

### Trends in anaemia prevalence by country and country income

In our analysis of trends and shifts in paediatric anaemia prevalence, we considered absolute and relative changes and average annual rates of reduction in the 21 EMR countries (**Table 1**). The analysis revealed reductions in most countries, but these reductions varied by country and national income. The overall prevalence among children in the EMR averaged 36.37% during the study period. From 2000 to 2019, the mean prevalence decreased notably from 40.56 to 34.25% – an absolute change of −6.31% and a relative change of -16%. This indicates general improvement in the treatment of anaemia among the population of this region (**Table 1**).

**Table 1:** Trends in mean anaemia prevalence in children aged 6–59 months in the Eastern Mediterranean Region by income group (2000–2019)

Income	Low income	Lower-middle income	Upper-middle income	High income	Total
2000					
*Mean*	58.76	41.75	35.05	26.67	40.56
*Interval*	33.3, 82.6	26.1, 61.7	32.2, 36.9	19.6, 39.6	19.6, 82.6
2005					
*Mean*	55.38	38.96	32.25	24.42	37.75
*Interval*	31.4, 81.7	23.0, 59.7	31.0, 33.5	17.9, 35.8	17.9, 81.7
2010					
*Mean*	53.28	36.68	28.50	22.45	35.23
*Interval*	30.5, 80.0	21.2, 59.0	27.5, 29.5	17.4, 30.5	17.4, 80.0
2015					
*Mean*	52.34	35.23	27.20	21.53	34.07
*Interval*	32.2, 79.9	21.8, 56.1	26.3, 28.4	18.6, 26.1	18.6, 79.9
2019					
*Mean*	51.98	35.00	28.00	22.02	34.25
*Interval*	32.9, 79.5	22.8, 53.0	26.6, 29.4	19.8, 24.3	19.8, 79.5
Total					
*Mean*	54.35	37.52	30.20	23.42	36.37
*Interval*	30.5, 82.6	22.2, 59.0	26.3, 29.4	18.6, 39.6	18.6, 82.6
Absolute change	−6.78	−6.75	−7.05	−4.65	−6.31
Relative change	−12.0%	−16.0%	−20.0%	−17.0%	−16.0%

In 2019, the prevalence of anaemia in children was found to exceed 40% in six countries, compared to seven countries in 2000. Over these 19 years, most countries achieved notable reductions in prevalence. Reductions ≥20% were reported in five countries, while six countries achieved reductions from 15 to 19%, five countries managed reductions from 11 to 14% and one nation reduced the prevalence by 5% (**Table 2**).

**Table 2:** Prevalence of paediatric anaemia across 21 Eastern Mediterranean Region countries by income level (2000–2019)

Countries	Income	Year	Prevalence%	Absolute change%	Relative change %	Average annual rate of reduction (95% CI)
Afghanistan	Low income	2000	51.5	−6.6	−13	−0.35 (−0.64, −0.05)
		2019	44.9			
Bahrain	High income	2000	26.2	−2.8	−11	−0.15 (−0.28, −0.02)
		2019	23.4			
Djibouti	Lower middle income	2000	61.5	−9.5	−15	−0.50 (−0.71, −0.29)
		2019	52			
Egypt	Lower middle income	2000	39.6	−7.4	−19	−0.39 (−0.56, −0.22)
		2019	32.2			
Iran	Lower middle income	2000	40.9	−14.4	−35	−0.76 (−0.93, −0.58)
		2019	26.5			
Iraq	Upper middle income	2000	36.9	−7.5	−20	−0.39 (−0.62, −0.17)
		2019	29.4			
Jordan	Lower middle income	2000	32.6	0.1	0	0.01 (−0.14, −0.15)
		2019	32.7			
Kuwait	High income	2000	20	−0.2	−1	−0.02 (−0.25, 0.22)
		2019	19.8			
Lebanon	Lower middle income	2000	26.1	−3.3	−13	−0.17 (−0.35, 0.01)
		2019	22.8			
Libya	Upper middle income	2000	33.2	−6.6	−20	−0.35 (−0.49, −0.20)
		2019	26.6			
Morocco	Lower middle income	2000	36	−5.6	−16	−0.29 (−0.41, −0.19)
		2019	30.4			
Oman	High income	2000	39.6	−15.3	−39	−0.81 (−0.93, −0.68)
		2019	24.3			
Pakistan	Lower middle income	2000	61.7	−8.7	−14	−0.45 (−0.60, −0.31)
		2019	53			
Qatar	High income	2000	25.2	−2.8	−11	−0.15 (−0.25, −0.05)
		2019	22.4			
Saudi Arabia	High income	2000	29.4	−7.6	−26	−0.40 (−0.60, −0.20)
		2019	21.8			
Somalia	Low income	2000	63.9	−12.1	−19	−0.64 (−0.76, −0.52)
		2019	51.8			
Sudan	Low income	2000	61.5	−10.7	−17	−0.56 (−0.76, −0.37)
		2019	50.8			
Syrian Arab Republic	Low income	2000	33.3	−0.4	−1	−0.02 (−0.18, 0.14)
		2019	32.9			
Tunisia	Lower middle income	2000	35.6	−5.2	−15	−0.27 (−0.47, −0.08)
		2019	30.4			
United Arab Emirates	High income	2000	19.6	0.8	4	0.04 (−0.06, 0.15)
		2019	20.4			
Yemen	Low income	2000	83.6	−4.1	−5	−0.22 (−0.30, −0.13)
		2019	79.5			

CI – confidence interval

**Figure 1:**
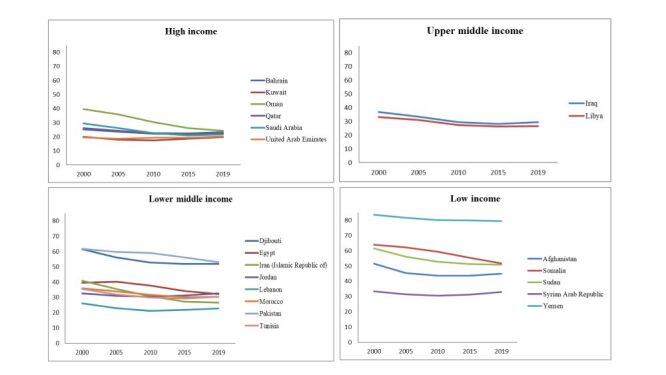
Prevalence of paediatric anaemia in the Eastern Mediterranean Region by income (2000–2019).

The changes in paediatric anaemia prevalence varied by country and income level (**Figure 1**). In agreement with our previous global analysis [14], this analysis identified a strong association (*P* < 0.001) between anaemia prevalence and country income. This significance persisted throughout the whole study period (2000–2019) and within each five-year interval (**Table 3**).

**Table 3:** Analysis of anaemia prevalence in Eastern Mediterranean Region children by income groups across different periods using Kruskal-Wallis H test

Prevalence of anaemia in children grouping variable: income	Kruskal-Wallis H	*P*-value*
All Periods 200 –2019	239.759	0.000
2000	10.023	0.018
2005	9.456	0.024
2010	11.376	0.010
2015	13.678	0.003
2019	14.063	0.003

*P < 0.05.

The prevalence remained the highest in low-income countries, which achieved a 12% average reduction from 58.76 to 51.98% in the study period. Although there was a decline in anaemia prevalence, it can be considered slow compared to the other income groups. For example, Yemen reported prevalences of 83.6 and 79.5% in 2000 and 2019, respectively – an absolute change of −4.1%, a relative change of −5% and an annual reduction of 0.22%. Sudan accomplished a considerable reduction in prevalence, from 61.5 to 50.8%, with an absolute change of −10.7% and a relative reduction of −17%. Their annual reduction rate was −0.56%. Among the low-income countries, Somalia achieved the best reduction in anaemia prevalence, from 63.9 to 51.8%, marking an absolute change of −12.1% and a relative reduction of −19%. Their annual reduction rate was −0.64% (**Table 2**).

Lower-middle-income countries reported significant decreases in anaemia prevalence, from 41.75% in 2000 to 35.0% in 2019. The mean reduction equalled a −16% relative change. Morocco and Tunisia showed improvement in the prevalence of anaemia. Tunisia decreased from 35.6% in 2000 to 30.4% in 2019, marking an absolute change of −5.2% and a relative change of −15%. Their annual reduction rate was −0.27%. Morocco reported a decrease from 36 to 30.4%. This marked a −5.6% absolute change and a −16% relative change. Their annual reduction rate was −0.29%. In this group, Iran achieved a large decrease in anaemia prevalence, from 40.9% in 2000 to 26.5% in 2019 – a −14.4% absolute change and a −35% relative change. Their annual reduction rate was −0.76% (**Table 2**).

Larger decreases in anaemia prevalence were reported by the upper-middle-income countries in the region. The prevalence decreased from 35.05% in 2000 to 28.0% in 2019, a -20% relative change. The prevalence in Libya decreased from 33.2% in 2000 to 26.6% in 2019. This was a −6.6% absolute change and a −20% relative change. The annual reduction was −0.35%. In the same group, Iraq reported a decrease from 36.9 to 29.4% from 2000 to 2019 – an absolute change of −7.5% and a relative change of −20%. The annual reduction was 0.39% (**Table 2**).

High-income countries accomplished a significant reduction in anaemia prevalence, from 26.67% in 2000 to 22.02% in 2019. Oman achieved the best reduction rate in the EMR, from 39.6% in 2000 to 24.3% in 2019 – a −15.3% absolute change and −39% relative change. The annual reduction was −0.81%. In the same group, Saudi Arabia reported a decrease from 29.4 to 21.8%, marking a −7.6% absolute change and −26% relative change. The annual reduction rate was −0.40%. On the other hand, another high-income country, the United Arab Emirates, experienced a small increase in anaemia prevalence, from 19.6% in 2000 to 20.4% – an absolute change of 0.8% and a relative change of 4%. The annual reduction was 0.04% (**Table 2**).

### Anaemia trends by five-year intervals

In 2000, the prevalence of anaemia was higher in low-income countries, at 58.76%, compared to 26.67% in high-income countries. By 2005, there had been a decrease throughout all income groups: 55.38% in low-income countries, 24.42% in high-income countries, and 38.96 and 32.25%, respectively, in lower-middle-income and upper-middle-income countries (**Table 1**).

From 2005–2010, the prevalence continued to decline in all income groups. The low-income group managed a decrease from 55.38 to 53.28%. The lower-middle-income group achieved a decrease from 38.96 to 36.68%, while the upper-middle-income countries achieved a notable reduction to 28.50%. High-income countries reduced their prevalence by up to 22.45%.

Although the rate of decline slowed in 2010–2015, there was still a continuous decrease in all income groups. The low-income group showed little improvement, at 52.34%. The lower-middle- and upper-middle-income groups reported decreases to 35.23% and 27.20%, respectively, while the high-income group achieved the most significant reduction, at 21.53% (**Table 1**).

From 2015 to 2019, all income groups continued to reduce anaemia. The low-income group showed a small decrease, at 51.98%. The lower-middle-income group achieved a greater decrease, at 35%, while the higher-middle-income group declined to 28.00% and the high-income group declined to 22.02%. The 2015–2019 period exhibited the smallest change compared to the previous periods (**Table 1**).

### 20-year behaviours of income groups

From 2000 to 2019, there was an overall decrease in the prevalence of anaemia from 40.56 to 36.37% – a −6.31 absolute change and a −16% relative change. The low-income group achieved a reduction from 58.76 to 51.98%, marking a −6.78 absolute change and a −12% relative change. The lower-middle-income group reduced their rate from 41.75 to 35%, which was a −6.75 absolute change and a −16% relative change. The upper-middle-income group reduced the rate from 35.05 to 28.00%, achieving a −7.05 absolute change and a −20% relative change. Finally, the high-income group reduced their rate from 26.67 to 22.02%, accomplishing a −4.65 absolute change and a −17% relative change (**Figure 1**).

### EMR trends *vs*. global trends (2000–2019)

**Figure 2:**
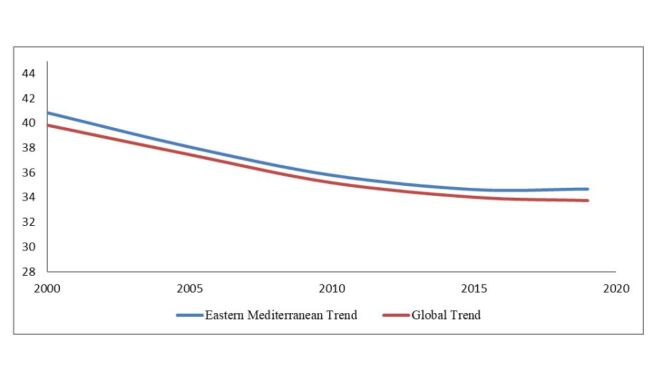
Comparison of trends in paediatric anaemia prevalence: global *vs*. EMR (2000–2019). The prevalence is always higher in the EMR despite a steady drop after 2015. EMR – Eastern Mediterranean Region.

In our previous study, global decreases in anaemia prevalence were examined [14]. These results can be usefully compared with the current study’s findings. The prevalence of anaemia steadily decreased between 2000 and 2019, both globally and in the EMR. In 2019, the prevalence of anaemia in the EMR was 34.6% greater than the global average (33.7%). Both trends demonstrated a gradual drop until 2015, when the global prevalence was 33.9% and the EMR’s rate was 34.6%. After 2015, the global level continued to decline (33.7%) until 2019, while the EMR trend plateaued at 34.7% in 2019. This represents a cumulative decrease of 6.2% between 2000 and 2019. Although there was a continual decrease, the EMR consistently exhibited a higher prevalence than the world as a whole (**Figure 2**).

## DISCUSSION

This study explored 20-year changes in anaemia prevalence among children aged 6–59 months in 21 countries in the EMR, correlating these developments with the national income levels of EMR countries. The overall trend was a gradual decline, but there were significant variances among and within socioeconomic categories. High levels of anaemia persist in low- and lower-middle-income nations, highlighting the complexity of the situation and suggesting that a high income alone is not sufficient to create change [17].

The prevalence of anaemia in the EMR dropped from 40.56% in 2000 to 34.25% in 2010–2019. The decline varied greatly across socioeconomic levels and countries. Prevalences were high (>70%) in low-income countries, such as Yemen. However, other countries, such as Somalia, demonstrated significant reductions from the previous round. High-income countries, such as Saudi Arabia and Oman, performed particularly well, while others, such as the UAE, demonstrated stasis or slight growth. In 2019, six countries in the region reported anaemia rates exceeding 40%, which is considered a significant public health concern by the WHO. Our study found a substantial connection (*P* < 0.001) between anaemia prevalence and income level. However, country-specific exceptions (Somalia, UAE) highlight the importance of other contextual factors [17,18].

Our findings align with those of global studies, such as the GBD study and Safiri et al., which discovered a gradual decrease in anaemia due to nutritional interventions [17,19]. However, after 2015, the EMR took a different path, as its progress plateaued. Compared to Southeast Asia and Latin America, where policies and governance have been more coordinated, the EMR’s high prevalence suggests shortcomings in implementation and follow-through [13,20]. This study fills a gap in the regional literature by examining long-term anaemia trends stratified by income in the EMR. This approach was not commonly used in previous research.

Slower decreases in anaemia in EMR nations may be linked to conflict, poor health systems and socioeconomic instability. Conflict, displacement and economic collapse in nations such as Yemen, Syria and Sudan have disrupted nutrition, food access and health care systems [17,21,22]. Some countries, such as Somalia, may yet see improved results due to targeted humanitarian aid, but poverty remains a concern [23]. Good governance, external support and focused programme implementation can be effective in low-income contexts [24].

Globally, anaemia prevalence declined more noticeably [14] than it did in the EMR setting. In 2019, the EMR’s prevalence remained higher than the global rate (34.25 *vs*. 33.7%). Southeast Asia and Latin America made significant improvements through improved public health nutrition programmes, political stability and coordinated regional efforts [25]. The plateau in the EMR since 2015 is a divergence that requires focused governmental attention. Regional instability and fragmented health systems may contribute to slower and unequal improvement rates in the EMR [26–28].

Anaemia in the EMR is influenced by cultural dietary patterns, environmental exposures and inequities in health care access, rather than just income or policy. Traditional meals in EMR countries often contain low amounts of haem iron and high levels of iron absorption inhibitors [29]. Inadequate infrastructure for water, hygiene and sanitation causes chronic infection and inflammation, exacerbating iron deficiency [30]. A lack of awareness of nutritional diversity, as well as cultural context, might also contribute to this condition [31]. Access to antenatal care and child health services is becoming increasingly disparate among countries [32].

Although the Eastern Mediterranean Nutrition Strategies 2010–2019 and 2020–2030 aim to minimise anaemia, the outcomes have been uneven [33]. While high-income nations have successfully implemented fortification and screening measures, low- and middle-income countries lack the necessary resources, political will and operational capacity to execute similar programmes [34]. Oman and Saudi Arabia demonstrate the positive impact of stability and proper funding on health programmes [35,36], whereas Yemen, Sudan and Syria highlight the vulnerability of health programming to ongoing crises [27]. The varied outcomes emphasise the importance of programme delivery and institutional capabilities [37].

This study’s key strength is the adoption of the 2022 World Bank income classification across the chosen time series. This strategy ensures consistency and comparability across times and countries, avoids reclassification issues and aligns with worldwide health metric guidelines [15,16]. It allows for a timely, policy-relevant analysis of long-term health patterns. A single classification allows policymakers to compare anaemia across communities based on current income levels rather than outdated classifications.

Interventions should take income and context into consideration. For example, low-income countries should implement basic measures, such as iron and folic acid supplementation [38], dietary fortification, maternal health outreach and mobile clinics [39]. Middle-income nations should focus on scaling fortification, nutrition education and improved prenatal care [40]. High-income countries should address dietary quality, conduct comprehensive screenings and modernise public health nutrition policy [41,42]. The effectiveness of food fortification varies by socioeconomic level; thus, it is important to consider regulatory enforcement, consumption patterns and public awareness [43,44].

However, the plateau that has persisted since 2015 suggests that it may be time to reassess current policies. Countries must scale up effective initiatives, particularly in underserved areas. Collaboration between the health, education and agriculture sectors is critical for success. Implementing real-time data, tailored actions and regional collaboration can accelerate results [45]. Regional progress should be influenced by lessons learned from Oman, Somalia and Saudi Arabia through strong government investment in health infrastructure and coordinated efforts with development partners [29,46]. A regional learning platform managed by the WHO could help disseminate effective models implemented in high-performing countries, thereby promoting sustainable and equitable progress [33].

### Limitations

Despite its contributions, this study also has limitations. We used secondary data from the WHO, which could have been subject to reporting bias and methodological inconsistency in different EMR countries. Each survey instrument may differ in quality and the data collection tools used, and estimation methods may vary, affecting comparability between countries and influencing the accuracy of trend estimates, particularly for countries with limited data infrastructure.

Additionally, the analysis did not consider major contextual determinants of anaemia prevalence, including welfare, food insecurity, political instability and cultural dietary customs, which could have affected the national results. Although we recognise the contributions of social and environmental determinants of health, our exclusive focus on national-level prevalence and income stratification limits our scope in capturing such dynamic and locally specific influences. These factors limit the generalisation of the findings to other countries/regions or conclusions

## CONCLUSIONS

This study identified a notable decrease in the prevalence of anaemia in children aged 6–59 months from 2000 to 2019 across the Eastern Mediterranean Region. However, that progress has been uneven: Low-income countries still report alarmingly high rates, while some high-income countries have stagnated or even seen increases. High anaemia rates were observed despite positive social determinants, such as high educational attainment and low poverty levels, suggesting that income alone is insufficient to reduce anaemia, and that additional structural and contextual factors need to be addressed.

The plateau seen since 2015 emphasises the need to reassess current interventions. To accelerate progress, countries must undertake context-specific, equity-focused strategies, especially in fragile and underserved settings. Key measures for moving forward include ensuring food security, broadening targeted supplementation, enhancing maternal and child health services and ensuring culturally appropriate nutrition education. Regional learning platforms could also facilitate the flow of knowledge and policies from better-performing to lagging countries.

To close gaps and realise meaningful and sustained reductions in childhood anaemia across the EMR, continued investment, innovation and cross-sector collaboration are paramount.
